# Clinical and Immunological Associations of Plasma Platelet-Activating Factor in Chronic Spontaneous Urticaria

**DOI:** 10.3390/biomedicines14081745

**Published:** 2026-08-02

**Authors:** Lāsma Lapiņa, Klinta Suhecka, Guna Ziedone, Nataļja Kurjāne

**Affiliations:** 1 Institute of Oncology and Molecular Genetics, Riga Stradiņš University, LV-1007 Riga, Latvia; klinta.suhecka@gmail.com (K.S.); natalja.kurjane@rsu.lv (N.K.); 2 Centre for Clinical Immunology and Allergology, Pauls Stradiņš Clinical University Hospital, LV-1002 Riga, Latvia; 3 Centre for Diagnosis and Treatment of Allergic Diseases, LV-1003 Riga, Latvia; guna.haitova@gmail.com

**Keywords:** platelet activating factor, chronic urticaria, biomarkers, disease progression, inflammation

## Abstract

**Background/Objectives**: Chronic spontaneous urticaria (CSU) is a heterogeneous immune-mediated condition involving multiple inflammatory pathways. The clinical utility of platelet-activating factor (PAF) as a biomarker in CSU remains insufficiently defined. This study aimed to evaluate plasma PAF levels in patients with CSU and to investigate their associations with clinical characteristics, disease activity, and laboratory and immunological parameters. **Methods**: This cross-sectional study included 205 adult patients diagnosed with CSU between January 2016 and May 2023. Clinical data, patient-reported outcome measures (PROMs), and extensive laboratory parameters were collected. Plasma PAF levels were measured using ELISA in 171 patients. Associations were analyzed using appropriate statistical methods. **Results**: The cohort was predominantly female (77.6%), with a mean age of 42.6 ± 15.3 years. Most patients had uncontrolled disease (76.9%). The median Urticaria Activity Score over 7 days (UAS7) score was 14, corresponding to mild disease activity. PAF levels differed significantly across clinical subgroups. Higher PAF levels were observed in patients with angioedema (alone or in combination with urticaria) compared to those with urticaria alone (*p* = 0.002), and in patients with predominantly nocturnal and early morning symptoms (*p* = 0.018). No associations were found between PAF levels and disease activity scores or treatment response. PAF levels showed positive correlations with age, age at symptom onset, IgM, IgG anti-IgE, HSP70, and the CD4/CD8 ratio (all *p* < 0.05). **Conclusions**: Plasma PAF levels are associated with specific clinical phenotypes, circadian symptom patterns, and selected immunological markers in CSU, suggesting that PAF may serve as a marker of underlying biological heterogeneity and immune activation. However, the present cross-sectional findings do not establish clinical utility, prognostic significance, or predictive relevance for treatment response, and these aspects require further longitudinal investigation.

## 1. Introduction

Chronic spontaneous urticaria (CSU) is a chronic inflammatory skin disease characterized by the recurrent occurrence of pruritic wheals, angioedema, or both for at least six weeks in the absence of identifiable external triggers [1,2,3,4]. It is primarily driven by activation and degranulation of skin mast cells, leading to the release of inflammatory mediators responsible for wheal formation, angioedema, and pruritus [4].

CSU is increasingly recognized as a heterogeneous immune-mediated condition involving multiple inflammatory pathways. Skin biopsies demonstrate infiltration of mast cells, basophils, eosinophils, and T lymphocytes, along with increased expression of adhesion molecules and pro-inflammatory cytokines [3,5]. These mechanisms contribute to persistent inflammation and may explain variability in disease activity, clinical presentation, and response to therapy among patients [5,6].

Although histamine is the principal mediator of pruritus via activation of histamine H1 receptors, a substantial proportion of patients show inadequate response to antihistamine therapy, suggesting involvement of additional mediators. These include prostaglandins, leukotrienes, neuropeptides, and platelet-activating factor (PAF) [7]. This has prompted increasing interest in non-histamine pathways that may contribute to disease persistence and therapeutic refractoriness.

PAF is a potent bioactive phospholipid involved in inflammation and immune regulation [8,9]. It is synthesized through two principal pathways. During inflammatory activation, PAF is generated predominantly from membrane phospholipids via the remodeling pathway, whereas the de novo pathway contributes mainly to basal constitutive PAF synthesis. PAF is produced by multiple immune cells, including mast cells, basophils, eosinophils, neutrophils, and macrophages, and exerts its effects via the platelet-activating factor receptor (PAFR), promoting leukocyte activation, mediator release, and increased vascular permeability [5,10,11]. These properties suggest that PAF may act as an amplifier of inflammatory responses in CSU [5,12].

Clinical evidence indicates that PAF may be associated with disease activity and treatment response in CSU. Elevated serum PAF levels, together with reduced activity of platelet-activating factor acetylhydrolase (PAF-AH), have been linked to increased disease severity and reduced responsiveness to antihistamine therapy [5,6,12]. Furthermore, PAF has been proposed as a potential predictor of antihistamine refractoriness [6].

Despite these observations, the clinical utility of PAF as a biomarker in CSU remains insufficiently defined. In particular, its relationship with clinical characteristics and laboratory markers of disease activity has not been fully elucidated. Given the need for reliable biomarkers to improve disease stratification and guide treatment decisions in CSU, further investigation of PAF is warranted.

This study aimed to evaluate plasma PAF levels in patients with CSU and to investigate their relationship with clinical characteristics, circadian symptom patterns, laboratory and immunological markers, and treatment response. In addition, selected markers of immune dysregulation, including HSP70, IgM, and the CD4/CD8 index, were assessed as exploratory indicators of danger-associated signaling, humoral immune activation, and alterations in cellular immune balance that may contribute to CSU pathogenesis.

## 2. Materials and Methods

### 2.1. Study Population

The study cohort was identified through the electronic medical records system of Pauls Stradiņš Clinical University Hospital, including patients diagnosed with CSU between January 2016 and May 2023. Additional participants were recruited from the Allergic Diseases Diagnosis and Treatment Center, a specialized referral centre for allergic diseases in Latvia.

CSU was diagnosed according to international guidelines [1,2,3,4]. Eligible participants were adults (≥18 years) with a confirmed diagnosis of CSU. Patients with chronic inducible urticarias, hereditary or bradykinin-mediated angioedema, angioedema associated with angiotensin-converting enzyme inhibitor use, and other alternative causes of recurrent wheals or angioedema were excluded. Patients were invited to participate during routine outpatient visits. After receiving detailed information about the study, all participants provided written informed consent.

Clinical interviews, completion of patient-reported outcome questionnaires, and blood sampling for PAF measurements were performed during the same study visit. Laboratory parameters retrieved from the national electronic medical information system (DataMed) were included if they had been obtained within one month of the study visit and were considered representative of the patients’ clinical status at inclusion.

Information regarding treatment status referred to ongoing treatment at the time of study participation and blood sampling, whereas treatment-response assessments were based on patients’ reported effectiveness of previous and/or current therapies according to their clinical history.

The study was approved by the Ethics Committee of Rīga Stradiņš University (Approval No. 2-PĒK-4/68/2023).

### 2.2. Clinical Data Collection

A total of 205 patients were included and evaluated by a board-certified allergologist. During study visits, participants completed validated disease-specific questionnaires, including the chronic urticaria quality of life questionnaire (CU-Q2oL), urticaria control test (UCT), urticaria activity score over 7 days (UAS7), urticaria severity score (USS), and angioedema-related instruments (AE-QoL, AAS, AECT), as well as a visual analogue scale (VAS). Venous blood samples for PAF measurements were collected during the same visit.

Demographic and clinical data were collected, including age, sex, education, occupational status, smoking, comorbidities, disease duration, symptom distribution, disease severity, circadian variation, triggers, and treatment history. Information regarding treatment status referred to ongoing treatment at the time of study participation and blood sampling, whereas treatment-response assessments were based on patients’ reported effectiveness of previous and/or current therapies. Treatment responses were classified as effective (complete symptom control), partially effective (symptom improvement with persistent residual symptoms), or ineffective (no clinically meaningful symptom improvement).

Laboratory parameters were retrieved from the national electronic medical information system (DataMed) and were included if available within one month of the study visit, thereby reflecting the patients’ clinical status at study inclusion.

### 2.3. Laboratory Data Acquisition

Laboratory test results, including leukocyte subpopulations (Leu, Neu, Ly, Mo, Eo, Ba), inflammatory markers (CRP, D-dimer, IL-6, RF, ASO, ECP), thyroid and metabolic parameters (TSH, FT4, PTH, vitamin D), immunoglobulins (IgG, IgA, IgM, IgE), complement components (C1 inhibitor concentration and activity, C3c, C4), and autoantibodies (IgG-anti-TPO, IgG-anti-TG, IgG-anti-TSH receptor, IgG-anti-C1q, IgG-anti-dsDNA, and anti-cardiolipin IgG/IgM/IgA, ANA, ENA, pANCA, and cANCA), as well as tumour markers (CA 19-9, CEA, CA15-3, CA125), were obtained from the Latvian electronic medical information system, DataMed. This system integrates laboratory and imaging data from multiple certified laboratories across the country, ensuring standardized and reliable measurements.

### 2.4. Sample Collection and Measurement of PAF

Study visits and blood sample collection were specifically organized for the purposes of the study and were generally scheduled within a relatively standardized daytime interval, approximately between 10:00 and 13:00. Venous blood samples were collected under standardized conditions. Plasma was obtained using EDTA tubes and centrifuged at 1500× *g* for 10 min. Samples were aliquoted and stored at −80 °C until analysis. PAF measurements were performed on 15 October 2025. Storage duration therefore varied depending on the date of sample collection. Repeated freeze–thaw cycles were avoided, and samples underwent a single thaw before analysis.

PAF concentrations were measured using a competitive enzyme-linked immunosorbent assay (ELISA) kit (Platelet Activating Factor ELISA Kit, Antibodies-online GmbH, Aachen, Germany; catalogue no. ABIN6969371) according to the manufacturer’s instructions. The assay had a detection range of 0.156–10 ng/mL and an analytical sensitivity of 0.094 ng/mL. According to the manufacturer, the intra-assay and inter-assay coefficients of variation were <8% and <10%. Plasma samples were analyzed in duplicate, and concentrations were calculated using standard curves. The effective upper reportable concentration in the original plasma samples was 20 ng/mL; values exceeding this limit were recorded as >20 ng/mL. All measurements were performed under standardized laboratory conditions using identical assay procedures and reagents to minimize potential batch effects.

PAF measurements were available for 171 of the 205 enrolled patients. Owing to resource constraints and the exploratory nature of the biomarker analyses, plasma PAF measurements could not be performed in all available samples. Therefore, analyses involving PAF were restricted to patients with available PAF measurements.

### 2.5. Measurement of HSP70 and Autoantibodies

Serum levels of HSP70 and anti-HSP70 antibodies were measured using commercially available ELISA kits (Enzo Life Sciences, Farmingdale, NY, USA; catalogue no. ADI-EKS-715; catalogue no. ADI-EKS-750) according to the manufacturer’s protocols.

IgG autoantibodies against FcεRIα and IgE were measured using in-house chemiluminescent ELISA. 384-well MaxiSorp plates were coated overnight at 4 °C with recombinant human FcεRIα (ACROBiosystems, Newark, DE, USA; Cat. No. FCA-H5228) or recombinant human IgE (Millipore/Sigma, Burlington, MA, USA; Cat. No. 401152) at a concentration of 1 µg/mL. After blocking, serum samples diluted 1:100 were added in duplicate and incubated for 1 h at room temperature. Bound IgG antibodies were detected using HRP-conjugated goat anti-human IgG antibodies (Jackson ImmunoResearch, West Grove, PA, USA; Cat. No. 109-035-098), followed by enhanced chemiluminescence substrate development and immediate signal measurement using a Victor plate reader (PerkinElmer, Shelton, CT, USA). As these assays were used for exploratory research purposes, no established clinical reference ranges or validated diagnostic cut-off values are currently available. Therefore, antibody levels were analyzed as continuous variables and patients were not categorized as antibody-positive or antibody-negative.

### 2.6. Statistical Analysis

Data distribution was assessed using the Shapiro–Wilk test. Continuous variables are presented as mean ± standard deviation (SD) or median (interquartile range, IQR), as appropriate. Categorical variables are expressed as counts and percentages.

Comparisons between groups were performed using Student’s *t*-test or one-way ANOVA for normally distributed data, and Mann–Whitney U test or Kruskal–Wallis test for non-normally distributed data. When global differences were identified using the Kruskal–Wallis test, post hoc pairwise comparisons were performed using Dunn’s test with Benjamini–Hochberg adjustment for multiple comparisons. Associations between variables were assessed using Spearman’s rank correlation.

Analyses were performed using available-case data; therefore, the number of observations varied according to the availability of individual clinical, laboratory, and questionnaire data.

To account for potential confounding, additional multivariable linear regression analyses were performed with plasma PAF concentration as the dependent variable. For immunological variables demonstrating significant associations with PAF in univariate analyses, separate regression models were constructed adjusting for age, sex, and disease duration.

Because several circadian symptom-pattern categories contained only a small number of patients, an exploratory adjusted analysis was additionally performed using a clinically meaningful grouping of circadian patterns into predominantly nocturnal symptoms (nighttime symptoms and symptoms predominantly occurring at night and in the morning) and other symptom patterns. Multivariable linear regression analysis was then performed with plasma PAF concentration as the dependent variable and age, sex, disease duration, and clinical presentation (urticaria only, angioedema only, or both) included as covariates.

Correlation analyses were considered exploratory and hypothesis-generating. Variables were selected based on their potential biological relevance to CSU pathogenesis and PAF-related inflammatory mechanisms and included demographic characteristics, disease-related variables, laboratory parameters, and selected immunological markers. Because of the exploratory nature of these analyses, no formal correction for multiple testing was applied, and the findings should be interpreted cautiously.

A two-sided *p*-value < 0.05 was considered statistically significant. All analyses were performed using Jamovi (version 2.7.5).

## 3. Results

### 3.1. Clinical and Disease Characteristics

The study included 205 patients with CSU, of whom 77.6% (*n* = 159) were female. The mean age was 42.6 ± 15.3 years. Detailed clinical characteristics and treatment patterns are presented in Table 1.

### 3.2. Disease Activity, Severity and Control

Disease activity, severity, and control were assessed using validated patient-reported outcome measures (PROMs), including VAS, UCT, UAS7, CU-Q2oL, AAS7, AECT-3mo, and AE-QoL. The results are summarized in Table 2.

Overall, the study population demonstrated a high disease burden and suboptimal disease control. Based on UCT scores, only 1.4% (*n* = 2) of patients had completely controlled CSU, 20.9% (*n* = 29) had well-controlled disease, and 77.7% (*n* = 108) had uncontrolled CSU.

The median UAS7 score was 14 (IQR 4–28), indicating overall mild disease activity. While 12.1% (*n* = 17) of patients were urticaria-free, 28.6% (*n* = 40) experienced severe disease.

Among patients with angioedema, disease control was generally poor, with only four patients achieving controlled disease according to AECT-3mo.

### 3.3. Plasma PAF Levels Across Patient Subgroups

PAF measurements were available for 171 patients included in the study. Patients with available PAF measurements and those without PAF data demonstrated comparable demographic and clinical characteristics. No statistically significant differences were observed regarding sex distribution, age, BMI, disease duration, disease activity scores, or phenotype distribution (Supplementary Table S2), suggesting that missing PAF measurements were unlikely to introduce substantial selection bias.

**Figure 1:**
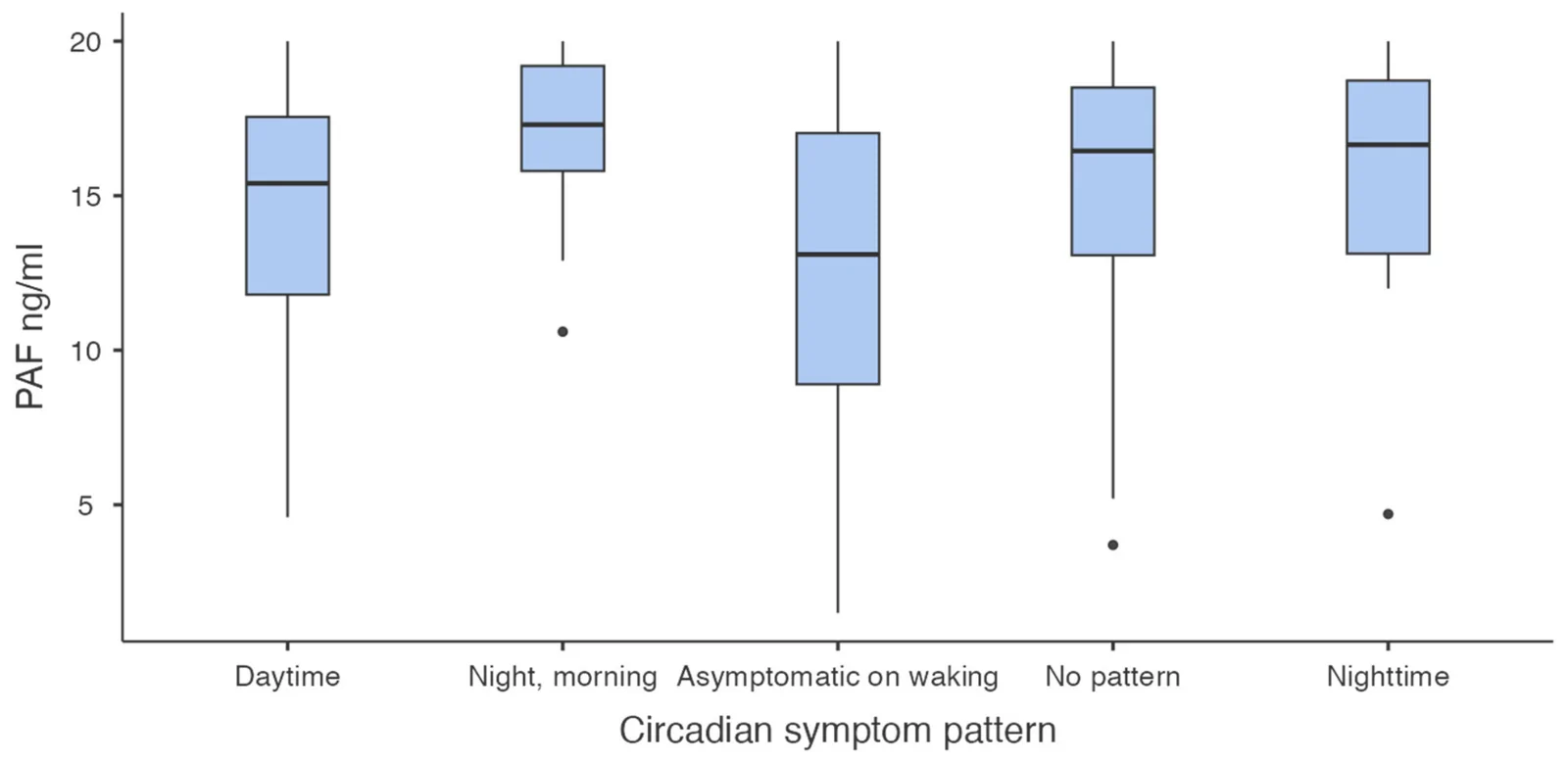
Plasma platelet-activating factor concentrations according to circadian symptom pattern. Black dots represent individual participants. Differences among groups were assessed using the Kruskal–Wallis test. Significant post hoc difference: predominantly night and early morning symptoms vs. asymptomatic on waking (adjusted *p* = 0.012). Higher levels were observed in patients with predominantly night and early morning symptoms compared with other circadian symptom pattern subgroups. Abbreviations: PAF—platelet-activating factor.

Significant differences in PAF levels were observed across circadian symptom patterns (*p* = 0.018) and clinical presentation (*p* = 0.002). The observed effect sizes were modest (η^2^ = 0.07 for both analyses), indicating that the magnitude of the associations was moderate despite reaching statistical significance. Post hoc pairwise comparisons using Dunn’s test with Benjamini–Hochberg adjustment demonstrated significantly higher PAF concentrations in patients with predominantly nocturnal and early morning symptoms (*n* = 25; median 17.3 [15.8–19.2] ng/mL) compared with patients who were asymptomatic on waking and developed symptoms mainly during the day (*n* = 28; adjusted *p* = 0.012), whereas no other pairwise differences remained statistically significant after adjustment for multiple comparisons (Figure 1). Because several circadian symptom subgroups contained only a small number of patients, these findings should be interpreted cautiously.

To account for potential confounding, an additional exploratory multivariable analysis adjusted for age, sex, disease duration, and clinical presentation was performed. After adjustment, the association between predominantly nocturnal symptom patterns and plasma PAF concentrations was attenuated and did not remain statistically significant.

**Figure 2:**
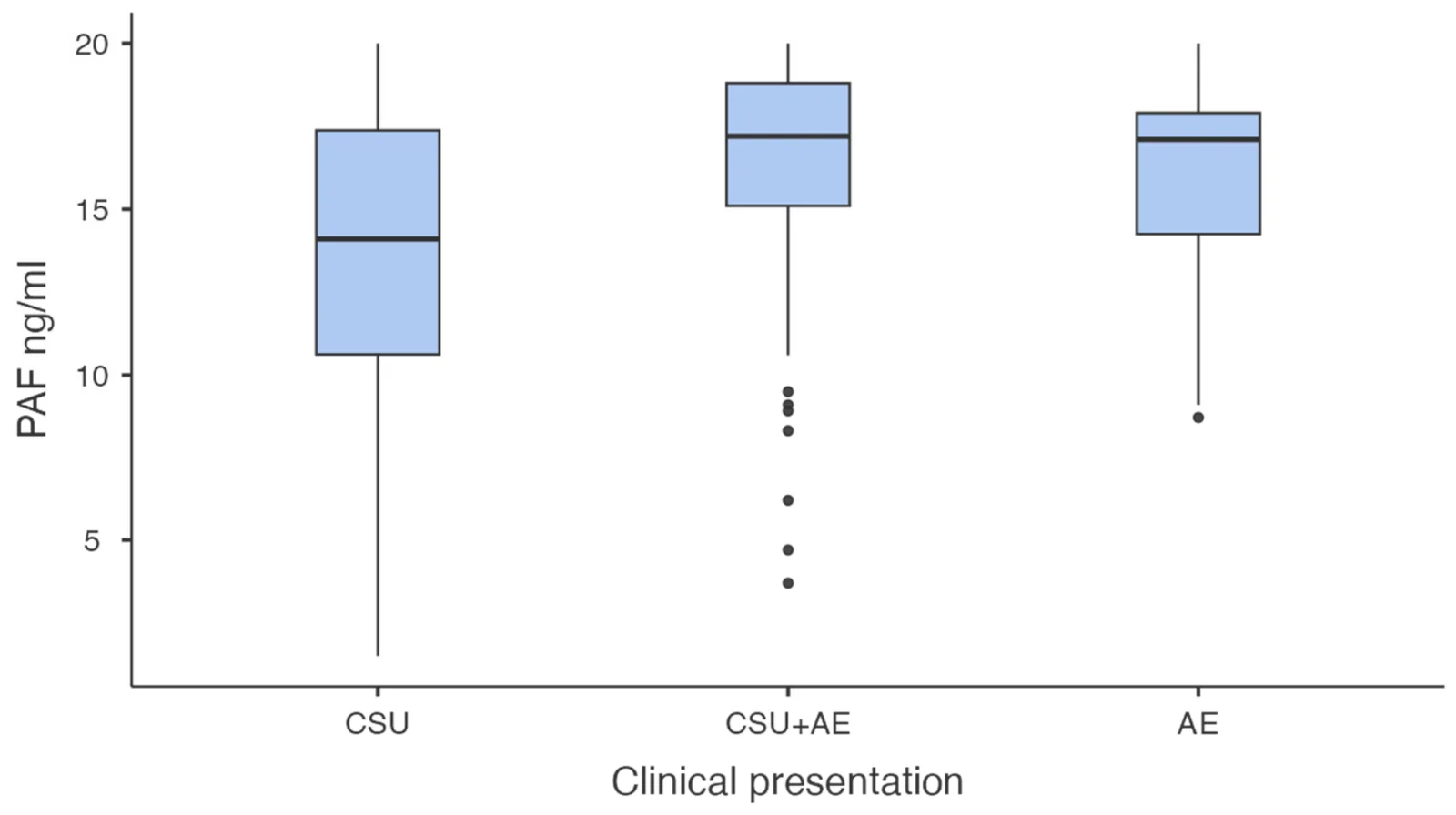
Plasma platelet-activating factor concentrations according to CSU clinical presentation. Black dots represent individual participants. Differences among groups were assessed using the Kruskal–Wallis test. Higher PAF levels were observed in patients with angioedema (alone or with urticaria) compared with those with urticaria alone. Significant post hoc difference: urticaria + angioedema vs. urticaria only (adjusted *p* = 0.003). Abbreviations: PAF—platelet-activating factor, CSU—chronic spontaneous urticaria, AE—angioedema.

PAF levels also differed across CSU clinical presentations (*p* = 0.002, η^2^ = 0.07). Post hoc pairwise comparisons demonstrated significantly higher PAF concentrations in patients with concomitant urticaria and angioedema (*n* = 77; median 17.2 [15.1–18.8] ng/mL) compared with patients with urticaria alone (*n* = 70; median 14.1 [10.6–17.4] ng/mL; adjusted *p* = 0.003). Although patients with isolated angioedema (*n* = 23; median 17.1 [14.3–17.9] ng/mL) also exhibited numerically higher PAF concentrations than patients with urticaria alone, this difference did not remain statistically significant after adjustment for multiple comparisons (adjusted *p* = 0.053). No significant difference was observed between patients with isolated angioedema and those with concomitant urticaria and angioedema (adjusted *p* = 0.631) (Figure 2).

No significant associations were observed between PAF levels and demographic or clinical variables, including sex, place of residence, allergic comorbidities, disease-triggering factors, accompanying symptoms, current treatment modalities, or treatment effectiveness (all *p* > 0.05).

Comparisons of plasma PAF levels across patient subgroups are presented in Table 3.

### 3.4. Correlation of PAF Levels with Clinical and Laboratory Parameters

Plasma PAF levels showed significant positive correlations with age (r = 0.202, *p* = 0.008), age at symptom onset (r = 0.208, *p* = 0.007), IgG anti-IgE (r = 0.186, *p* = 0.015), IgM (r = 0.215, *p* = 0.012), HSP70 (r = 0.171, *p* = 0.029), and the CD4/CD8 index (r = 0.369, *p* < 0.001). To account for potential confounding, additional multivariable linear regression analyses adjusted for age, sex, and disease duration were performed. Associations between PAF levels and IgM (adjusted β = 1.17, *p* = 0.007) and the CD4/CD8 ratio (adjusted β = 1.56, *p* = 0.003) remained statistically significant. In contrast, the associations with IgG anti-IgE and HSP70 were attenuated and were no longer statistically significant after adjustment.

No significant correlations were observed with disease duration, BMI, PROMs, or other laboratory parameters (all *p* > 0.05).

Correlations between plasma PAF levels and clinical and laboratory parameters are shown in Table 4.

## 4. Discussion

This study shows that plasma PAF levels are associated with clinical characteristics and selected immunological markers in CSU, supporting a role for PAF as a marker of immune activation and disease heterogeneity.

PAF is a pro-inflammatory phospholipid mediator involved in allergic inflammation and immune signaling and is produced by multiple cell types relevant to CSU pathogenesis, including mast cells and basophils [1,2,3]. Given its role in inflammatory processes, PAF has been explored as a biomarker in chronic inflammatory diseases [1,4].

CSU is a heterogeneous disease with distinct autoimmune endotypes, including type I (autoallergic) and type IIb (autoimmune) CSU, defined by different autoantibody profiles and immune mechanisms [5,6,7]. In this context, the observed correlation between PAF levels and anti-IgE IgG antibodies may suggest a possible association with autoimmune features of CSU. Some of the characteristics, such as predominantly nocturnal symptoms, eosinopenia, and lower IgA levels, have previously been reported in association with type IIb autoimmune CSU [13,14,15]. However, because the complete diagnostic criteria for type IIb autoimmune CSU, including functional autoantibody testing and comprehensive endotype characterization, were not systematically evaluated in the present study, these observations should be regarded as exploratory and hypothesis-generating. Therefore, no definitive conclusions regarding a specific association between plasma PAF concentrations and type IIb autoimmune CSU can be drawn.

We also identified associations between PAF levels and IgM antibodies, HSP70, and the CD4/CD8 ratio. Although direct evidence linking these markers to PAF in CSU is limited, these findings may reflect broader immune dysregulation. IgM autoantibodies have been described in CSU, although their specificity remains unclear [8], while alterations in the CD4/CD8 ratio and increased HSP70 levels have been associated with chronic immune activation and inflammatory stress responses [9,10]. These observations support the concept that PAF may be linked to multiple overlapping immune pathways rather than a single pathogenic mechanism.

To account for potential confounding, additional analyses adjusted for age, sex, and disease duration were performed. After adjustment, the associations of PAF with IgM and the CD4/CD8 ratio remained statistically significant, suggesting that these relationships may be relatively independent of major demographic and disease-related factors. Absolute CD4 and CD8 counts were unavailable and therefore it was not possible to determine which lymphocyte subset primarily contributed to the observed association. Conversely, the associations with IgG anti-IgE and HSP70 were no longer statistically significant after adjustment, indicating that these correlations may have been influenced, at least in part, by confounding factors.

In a large study by Ulambayar et al., PAF and PAF-acetylhydrolase levels were evaluated in CSU, with no association observed with age or disease activity, although correlations with metabolic parameters and antihistamine refractoriness were reported [11]. In contrast, we observed positive associations between PAF levels and age, age at symptom onset, circadian symptom patterns, and clinical presentation, but not with antihistamine response. These differences may be explained by differences in population characteristics (e.g., demographic or clinical profiles), variations in study design and sample size, as well as underlying biological heterogeneity of CSU.

Plasma PAF concentrations were not associated with current treatment status, treatment modalities, or treatment effectiveness, including response to antihistamines and omalizumab. This suggests that the observed associations between PAF and specific clinical characteristics are unlikely to be explained solely by ongoing therapy. The limited number of patients receiving advanced therapies, particularly ciclosporin, should be considered when interpreting these findings.

Consistent with previous reports demonstrating elevated PAF levels in CSU and in severe allergic reactions [11,12,13], we found higher PAF levels in patients with angioedema, either alone or in combination with urticaria, compared with those with urticaria alone. This pattern suggests that increased PAF levels may be associated with distinct clinical manifestations of CSU rather than overall disease severity. Given the established role of PAF in vascular permeability and inflammatory signaling, the observed association with angioedema may reflect more pronounced involvement of deeper tissues and increased plasma extravasation. These findings warrant further investigation into the potential role of PAF as a biomarker and possible therapeutic target in specific CSU phenotypes.

Self-reported time-of-day symptom patterns are a recognized feature of CSU, with symptoms often worsening during the evening and night [14,15,16,17,18]. In our study, higher PAF levels were observed in patients with predominantly nocturnal and early morning symptoms, suggesting a potential association between PAF concentrations and symptom timing. However, because endogenous circadian rhythms were not directly assessed, no conclusions regarding circadian regulation of PAF can be drawn.

Although direct evidence for circadian regulation of PAF synthesis is limited [19,20,21], indirect data suggest that PAF-related pathways may potentially be influenced by mechanisms involved in temporal immune regulation [20,21,22]. The observed associations may reflect differences in mast cell activity, mediator release, or other immunological processes associated with symptom timing rather than direct evidence of circadian regulation.

However, the observed association between self-reported time-of-day symptom patterns and PAF concentrations did not persist after adjustment for demographic and clinical factors. This finding suggests that the initial association may partly reflect differences in patient characteristics and disease phenotype rather than an independent relationship between circadian subgroups and PAF levels. Because of the relatively small numbers of patients in several time-of-day symptom pattern subgroups, these adjusted analyses should be considered exploratory and interpreted cautiously.

Despite the high disease burden observed in our cohort, PROMs did not correlate with PAF levels. This may suggest that circulating PAF reflects biological processes that are not directly aligned with patients’ subjective perception of symptoms. Alternative explanations should also be considered, including limited statistical power, variability in symptom assessment and blood sampling timing, treatment-related effects, and biological variability in circulating PAF concentrations. Patient-reported outcomes are influenced by multiple factors, including symptom perception, psychological burden, and individual variability, whereas PAF may capture specific pathophysiological processes that do not necessarily translate proportionally into perceived disease severity.

Several limitations should be considered that may have influenced the observed associations. Although the overall sample size was adequate, subgroup analyses were limited by unequal group sizes and small numbers in some subgroups, which may have affected the robustness of the findings. In addition, the sex distribution was unbalanced. Some clinical data were based on patient-reported history, and not all laboratory parameters were available for all participants. Several laboratory and questionnaire variables were unavailable for a proportion of participants because these investigations were performed according to clinical indications, differed across treating centres, or were not completed by all participants. Consequently, complete-case analyses could not be performed and the possibility of bias related to non-random missing data cannot be excluded. PAF levels were assessed at a single time point, precluding evaluation of their temporal dynamics. Although study visits and blood sample collection were specifically organized for research purposes and were generally conducted within a relatively standardized daytime interval (approximately 10:00–13:00), exact sampling times were not systematically recorded and therefore could not be incorporated into the analyses. Residual confounding related to sampling time cannot be completely excluded. No direct assessments of endogenous circadian rhythms, such as repeated 24 h sampling, sleep–wake measurements, melatonin, cortisol, or clock-related biomarkers, were performed. Therefore, findings related to symptom timing should be interpreted as associations with self-reported time-of-day symptom patterns rather than evidence of circadian regulation.

PAF measurements were available for only a subset of the study population (*n* = 171), primarily because of resource limitations related to the exploratory biomarker analyses. In addition, several subgroup analyses involved relatively small numbers of participants, particularly in some circadian symptom pattern subgroups, and these findings should therefore be interpreted cautiously and considered exploratory.

The marked predominance of female patients may also influence the generalizability of our findings. However, CSU is known to affect women considerably more frequently than men, and a similar female predominance has been consistently reported in epidemiological and clinical studies [6,7]. Therefore, the sex distribution observed in our cohort generally reflects the recognized demographic characteristics of CSU populations. Because of the substantially lower prevalence of CSU among men, establishing sex-balanced subgroups was not feasible without compromising sample size and statistical power.

Healthcare provision in Latvia is highly centralized, and the management of patients with CSU is predominantly concentrated within major tertiary referral centres providing specialized care for these patients nationwide. Recruitment from these centres likely reflects the national organization of CSU care rather than selection from a narrowly defined subgroup of patients with particularly severe disease.

Participation in the study may have been more appealing to patients with greater disease burden and persistent symptoms, potentially introducing volunteer bias. This may partly explain the prolonged and highly variable disease duration observed in our cohort, as patients referred to specialist centres frequently represent individuals with more severe, persistent, or difficult-to-treat disease [5,6,17]. The study population also included recently diagnosed patients as well as individuals with longstanding disease, thereby representing a broad spectrum of disease duration and clinical presentation.

Another limitation of the present study is the absence of a healthy control group. We were unable to determine whether circulating PAF levels are elevated in CSU compared with healthy individuals. The primary aim of this study was not to compare CSU patients with healthy subjects but rather to investigate the clinical and immunological correlates of PAF within a well-characterized CSU cohort. Therefore, our findings should be interpreted as associations within CSU patients and do not allow conclusions regarding disease-specific elevation of PAF.

Because of the cross-sectional design of this study, the present findings do not permit conclusions regarding the prognostic value or clinical utility of PAF as a predictive biomarker. Residual confounding from unmeasured clinical and biological factors cannot be excluded. The observed associations should therefore be considered hypothesis-generating and require confirmation in prospective longitudinal and mechanistic studies.

## 5. Conclusions

Plasma PAF levels were associated with clinical phenotype, self-reported time-of-day symptom patterns, and selected immunological markers in CSU, suggesting a potential role for PAF in disease heterogeneity and immune activation.

Higher PAF levels in patients reporting predominantly night and early morning symptoms, as well as in those with angioedema, point to associations with specific clinical phenotypes rather than overall disease severity.

The absence of correlations with patient-reported outcomes suggests that PAF may reflect underlying biological processes not fully captured by subjective measures.

However, owing to the cross-sectional design, the absence of a healthy control group, and single-time-point measurements, no conclusions can be drawn regarding the diagnostic, prognostic, predictive, or therapeutic utility of PAF. The observed associations should be considered hypothesis-generating and warrant confirmation in prospective longitudinal and mechanistic studies to clarify the potential role of PAF in CSU.

## Figures and Tables

**Table 1:** Clinical and demographic characteristics of the study population (*n* = 205).

Sex, *n* (%)	
Female	159 (77.6)
Male	46 (22.4)
Age, years (Mean, SD)	42.6 (15.3)
Higher education, *n* (%)	118 (57.6)
Place of residence, *n* (%)	
Urban	181 (88.4)
Rural	24 (11.7)
BMI, Median (IQR)	25.5 (21.6–30.9)
Smoking status, *n* (%)	
Non-smoker	113 (55.1)
Smoker	59 (28.8)
Former smoker	33 (16.1)
Clinical presentation, *n* (%)	
Urticaria only	81 (39.5)
Urticaria + angioedema	98 (47.8)
Angioedema only	26 (12.6)
Allergic comorbidity, *n* (%)	94 (45.8)
Duration of CSU, months	Mean (SD) 57.4 (71.8)
Age of symptom onset, years	Mean (SD) 36.1 (15.2)
Circadian symptom pattern, *n* (%)	
No circadian pattern	89 (43.4)
Only daytime	37 (18.0)
Only nighttime	11 (5.4)
Mostly at night and in the morning	35 (17.1)
Asymptomatic on waking	33 (16.1)
Accompanying symptoms, *n* (%)	128 (62.4)
Disease-provoking factors, *n* (%)	156 (76.1)
Therapy with antihistamines, *n* (%)	191 (93.2)
Therapy with omalizumab, *n* (%)	56 (27.3)
Therapy with cyclosporine, *n* (%)	8 (3.9)

Abbreviations: BMI—body mass index, CSU—chronic spontaneous urticaria, SD—standard deviation, IQR—interquartile range.

**Table 2:** Patient-reported outcome measures (PROMs).

Variable	*n*	Median (IQR)
VAS	139	8 (2–10)
UCT	139	8 (5.5–11)
UAS7	139	14 (4–28)
CU-Q2oL	139	34 (21–51)
AAS7	20	10.0 (0–78.8)
AECT-3mo	20	6.00 (3.75–9)
AE-QoL	19	38.2 (22.8–77.2)

The availability of PROMs varied according to questionnaire applicability and completion. Angioedema-specific questionnaires were completed only by patients with isolated mast cell-mediated angioedema (without concomitant wheals), whereas urticaria-specific questionnaires were completed only by patients presenting with urticaria manifestations. Abbreviations: IQR—interquartile range, VAS—visual analogue scale, UCT—urticaria control test, UAS7—urticaria activity score over 7 days, CU-Q2oL—chronic urticaria quality of life questionnaire, AAS7—angioedema activity score over 7 days, AECT-3mo—angioedema control test over 3 months, AE-QoL—angioedema quality of life questionnaire.

**Table 3:** Plasma PAF levels across selected patient subgroups.

Variable	Group			*n*	Median (IQR)	Effect Size	*p*
Sex	Female			132	16.3 (12.1–18.6)	r = 0.01	0.887
	Male			39	16.3 (13.3–18.3)		
Place of residence	Urban			151	16.0 (12.3–18.3)	r = 0.09	0.250
	Rural			20	16.7 (13.9–18.6)		
Allergic comorbidity	No			85	15.8 (11.9–17.9)	r = 0.09	0.241
	Yes			85	16.8 (13–18.7)		
Disease-triggering factors	No			51	16.0 (11.8–18.3)	r = 0.04	0.612
	Yes			119	16.5 (11.8–18.3)		
Circadian symptom pattern ^†^	No circadian pattern			78	16.4 (13.1–18.5)	η^2^ = 0.048	**0.018**
	Only daytime			6	15.4 (11.8–17.5)		
	Only nighttime			8	16.6 (13.1–18.7)		
	Mostly at night and in the morning			25	17.3 (15.8–19.2)		
	Asymptomatic on waking			28	13.1 (8.9–17.0)		
Response to antihistamine treatment	Effective			99	16.3 (13.0–18.4)	η^2^ = 0.017	0.223
	Ineffective			45	17.1 (11.9–18.6)		
	Partially effective			14	16.1 (11.8–17.5)		
Response to omalizumab treatment	Effective			31	16.9 (11.6–17.9)	η^2^ = 0.011	0.351
	Ineffective			9	17.9 (15.4–18.5)		
	Partially effective			8	13.7 (10.2–16.6)		
Clinical presentation ^†^	Urticaria only			70	14.1 (10.6–17.4)	η^2^ = 0.061	**0.002**
	Angioedema only			23	17.1 (14.3–17.9)		
	Urticaria + angioedema			77	17.2 (15.1–18.8)		
Accompanying symptoms	No			62	14.8 (11.5–18.7)	r = 0.07	0.359
	Yes			108	16.6 (13.5–18.0)		
Current treatment	Any antihistamine ^††^	108	16.6 (13.1–18.6)	0.077			
	Omalizumab	26	16.4 (10.6–18.3)	0.494			
	Cyclosporin	2	13.8 (11.7–16.0)	0.729			
	No current treatment	37	15.7 (11.8–18.0)	0.47			

Note: Plasma PAF measurements were available for 171 patients. The number of observations varies across subgroups because of missing data for individual variables; therefore, subgroup totals do not necessarily sum to 171. Continuous variables are presented as median (IQR). PAF concentrations were compared using the Mann–Whitney U test for two-group comparisons and the Kruskal–Wallis test for comparisons involving more than two groups. Post hoc pairwise comparisons were performed using Dunn’s test with Benjamini–Hochberg adjustment for multiple comparisons. Treatment categories were not mutually exclusive, and individual patients could receive more than one therapy simultaneously. Abbreviations: PAF—platelet-activating factor; IQR—interquartile range. ^†^ Significant pairwise differences are described in the Section 3. ^††^ Includes cetirizine, bilastine, quifenadine, rupatadine, desloratadine, levocetirizine, loratadine, clemastine, and chloropyramine.

**Table 4:** Correlation of PAF levels with clinical and laboratory parameters.

Variable	*n*	r	*p*-Value
CD4/CD8 index	78	0.369	<0.001
IgM	135	0.215	0.012
Age at symptom onset	170	0.208	0.007
Age	171	0.202	0.008
IgG anti-IgE	171	0.186	0.015
HSP70	163	0.171	0.029

Note: Correlations were calculated only among participants with available plasma PAF measurements and corresponding paired clinical or laboratory data; therefore, the number of observations varied between analyses. Only statistically significant correlations are shown. Full results are available in Supplementary Table S1. Abbreviations: CD4/CD8, CD4-positive to CD8-positive T-cell ratio; IgM, immunoglobulin M; IgG anti-IgE, immunoglobulin G anti-immunoglobulin E autoantibodies; HSP70, heat shock protein 70.

## Data Availability

All data generated or analyzed during this study are presented within this article. Additional information is available upon reasonable request by contacting the corresponding author.

## Supplementary Materials

The following supporting information can be downloaded at: https://www.mdpi.com/article/10.3390/biomedicines14081745/s1, Table S1: Correlation of PAF levels with clinical and laboratory parameters; Table S2: Comparison of patients with and without available PAF measurements.

## Abbreviations

The following abbreviations are used in this manuscript:

AECT-3mo	Angioedema control test over 3 months
AAS	Angioedema activity score over 7 days
AE-QoL	Angioedema quality of life questionnaire
BMI	Body mass index
CSU	Chronic spontaneous urticaria
CU-Q2oL	Chronic urticaria quality of life questionnaire
ELISA	Enzyme-linked immunosorbent assay
IQR	Interquartile range
PAF	Platelet-activating factor
PAF-AH	Platelet-activating factor acetylhydrolase
PAFR	Platelet-activating factor receptor
PROMs	Patient-reported outcome measures
SD	Standard deviation
UAS7	Urticaria activity score over 7 days
UCT	Urticaria control test
VAS	Visual analogue scale
