# Supplementary material for: Clinical and Immunological Associations of Plasma Platelet-Activating Factor in Chronic Spontaneous Urticaria
Source: Biomedicines. 2026 Aug 2;14(8):1745. doi: 10.3390/biomedicines14081745 (PMC13509238; doi:10.3390/biomedicines14081745)
Supplement: Supplementary file 1 [file biomedicines-14-01745-s001.zip › biomedicines-4427153-supplementary.pdf]

SUPPLEMENTARY

Table S1. Correlation of PAF levels with clinical and laboratory parameters

	PAF levels		
**Variable**	**n**	**r**	**p**
Age	171	0.202	**0.008**
BMI	170	0.139	0.114
Age of symptom onset	170	0.208	**0.007**
Duration of CSU	170	-0.004	0.958
VAS	114	0.050	0.596
UCT	114	0.064	0.499
UAS7	114	-0.080	0.396
CU-Q2oL	114	-0.009	0.925
AAS7	17	0.451	0.069
AECT-3mo,	17	-0.335	0.188
AE-QoL	16	0.460	0.073
IgG-anti-FcεRI	171	-0.050	0.514
IgG-anti-IgE	171	0.186	**0.015**
AntiHSP70 IgG/A/M	165	0.152	0.051
HSP10	163	0.045	0.561
HSP70	163	0.171	**0.029**
IL-10	170	0.062	0.422
IL-33	169	0.094	0.226
IL-17A	171	0.065	0.399
Leu	170	0.013	0.866
Neu	170	0.044	0.567
Ly	170	-0.046	0.553
Mo	170	0.036	0.638
Ba	170	-0.039	0.614
Eos	170	-0.023	0.763
CRP	170	0.069	0.370
D-dimers	98	-0.004	0.968
TSH	166	-0.036	0.650
FT4	110	-0.145	0.133
Vitamin D	137	0.039	0.655
IgG	137	0.066	0.443
IgA	136	0.160	0.063
IgM	135	0.215	**0.012**
IgE	159	-0.070	0.379
C1 inhibitor	44	-0.116	0.455
C3c	123	0.090	0.324
C4	131	-0.009	0.923
CD4/CD8 index	78	0.369	**<0.001**
CD19	78	0.042	0.718
CD3	78	-0.042	0.715

Serum tryptase	7	0.286	0.556
RF	121	0.008	0.934
ASO	66	0.132	0.289
ECP	19	0.198	0.416

Note: Correlation analyses were performed using available-case data. The reported sample sizes represent the number of participants with both available plasma PAF measurements and corresponding paired clinical or laboratory parameters, therefore, the number of observations varied between analyses. Abbreviations: AAS – angioedema activity score over 7 days, AECT-3mo – angioedema control test over 3 months, AE-QoL – angioedema quality of life questionnaire, ASO – antistreptolysin O, Ba – basophils, BMI – body mass index, CRP – C-reactive protein, CSU – chronic spontaneous urticaria, CU-Q2oL – chronic urticaria quality of life questionnaire, ECP – eosinophil cationic protein, Eos – eosinophils, HSP10 – heat shock protein 10, HSP70 – heat shock protein 70, IgA – immunoglobulin A, IgE – immunoglobulin E, IgG – immunoglobulin G, IgM – immunoglobulin M, IL10 – interleukin 10, IL17A – interleukin 17A, IL33 – interleukin 33, Leu – leukocytes, Ly – lymphocytes, Mo – monocytes, Neu – neutrophils, PAF – platelet-activating factor, RF – rheumatoid factor, TSH – thyroid-stimulating hormone, UAS7 – urticaria activity score over 7 days, UCT – urticaria control test, VAS – visual analogue scale.

Table S2. Comparison of patients with and without available PAF measurements

**Variable**	**PAF available (n = 171)**	**PAF unavailable (n = 34)**	**p-value**
Female sex, n (%)	132 (77.2%)	28 (82.4%)	0.63
Age, years	40.0 (30.5–54.0)	43.0 (28.0–52.0)	0.96
BMI, kg/m²	25.3 (21.4–30.9)	27.0 (22.7–31.6)	0.42
Disease duration, months	31.0 (15.8–72.0)	36.0 (11.0–69.0)	0.92
UCT	8.0 (5.0–11.0)	8.0 (6.0–11.0)	0.86
UAS7	15.0 (4.0–29.0)	14.0 (3.0–22.0)	0.44
AAS7*	5.0 (0–35.0)	77.0 (46.0–80.5)	0.23
Urticaria only, n (%)	70 (40.9%)	11 (32.4%)	
Urticaria and angioedema, n (%)	78 (45.6%)	21 (61.8%)	
Angioedema only, n (%)	23 (13.5%)	2 (5.9%)	0.31†

Data are presented as median (IQR) unless otherwise indicated. Continuous variables were compared using the Mann–Whitney U test. Categorical variables were compared using Fisher's exact test or Pearson's χ^2 test, as appropriate.

* AAS7 data were available only for a limited number of patients and should therefore be interpreted with caution.

† p-value for overall phenotype distribution.
